# Histologic differences between in situ and embolized carotid web thrombi: a case report

**DOI:** 10.1186/s12883-021-02428-w

**Published:** 2021-10-13

**Authors:** Qun Gao, Shen Hu, Ximeng Yang, Junjie Wang, Jun Lu, Daming Wang

**Affiliations:** 1 Department of Neurosurgery, Beijing Hospital, National Center of Gerontology, No.1 DaHua Road, Dong Dan, Beijing, 100730 People’s Republic of China; 2 Graduate School of Peking Union Medical College, Beijing, China

**Keywords:** Carotid web, Acute ischemic stroke, Histopathology, In situ thrombus, Embolized thrombus

## Abstract

**Background:**

The significance of carotid webs (CaWs) in ischemic stroke is becoming acknowledged. Histological features of clot composition in situ and secondary cerebrovascular embolized thrombi caused by CaW have not been described concurrently. Understanding clots’ histological composition is essential for understanding the pathophysiology of clot formation in CaW.

**Case presentation:**

A 50-year-old male patient with acute ischemic stroke, which was believed to be caused by ipsilateral CaW, was admitted to the hospital. Mechanical thrombectomy was used to retrieve thromboemboli from the middle cerebral artery. One month thereafter, the patient underwent carotid endarterectomy, and in situ CaW thrombi were retrieved. Histological analysis by hematoxylin and eosin staining revealed that histopathologic embolized thrombi appeared as typical mixed thrombi, 46.03% fibrin/platelet ratio, 48.12% RBCs, and 5.85% white blood cells. In situ thrombi had a higher fibrin/platelet ratio (68.0%), fewer RBCs (17.2%), and 14.8% white blood cells.

**Conclusion:**

The histopathology of large vessel occlusion stroke embolized thrombi by CaW is similar to that of other stroke etiologies. However, the clot composition of embolized thrombi significantly differs from that of in situ thrombi. CaW’s in situ thrombi showed predominantly fibrin, and embolized thrombi had equivalent contents of red blood cells and fibrin/platelets. Histopathological differences between in situ and embolized thrombi suggest new research directions for the etiology of embolization. Further studies are required to confirm these results.

## Background

A carotid web (CaW) is described as a rare focal intimal variant of fibromuscular dysplasia and implicated in acute ischemic stroke [1]. Several studies have demonstrated a high CaW prevalence among young adult patients with cryptogenic recurrent stroke [2–4]. Blood stasis of the rostral aspect of the CaW is hypothesized to later lead to thrombus formation and thromboembolic stroke [5]. Evidence of in situ thrombosis is observed in 12–29% of patients with stroke due to CaW [1, 6]. Although clots in situ CaW or secondary cerebrovascular embolized thrombi caused by CaW have been histologically described, in situ thrombi and clots of secondary thromboembolism have not been previously reported in the same CaW patient. Thus, we compared the histopathology of thrombi in situ CaW and cerebrovascular thromboembolism secondary to CaW. This report aimed to assess whether histopathological compositional differences exist between CaW thrombi in situ and embolized ones.

## Case presentation

**Fig. 1:**
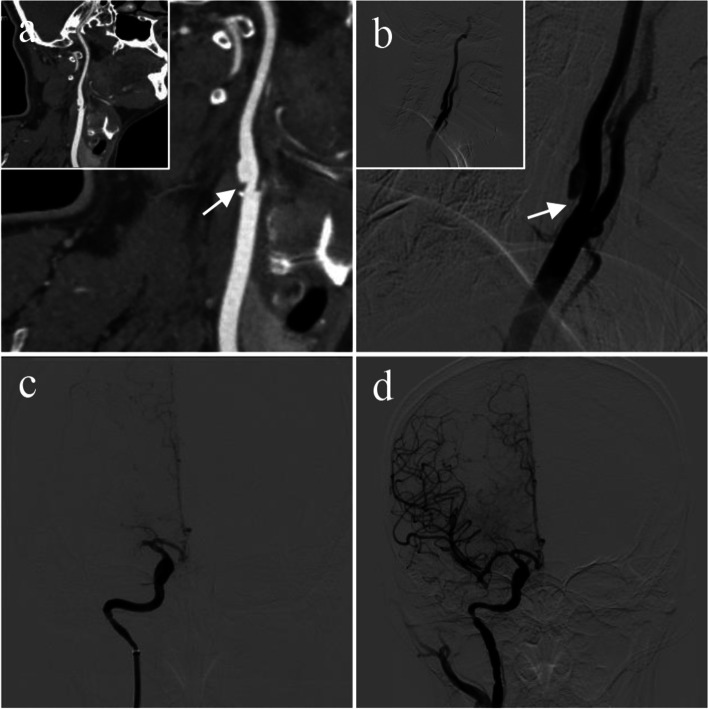
**a**, **b** CTA and DSA images of carotid artery webs showing a thin, membrane-like structure (white arrow) in the carotid bulb. **c** A right internal carotid angiogram demonstrating MCA occlusion. **d** Mechanical thrombectomy achieved recanalization of the right MCA

A 50-year-old male patient was admitted to the hospital because of left limb weakness and slurred speech for 2 h. Physical examination showed normal consciousness, gaze palsy, left central facial and glossal palsy, hypoesthesia of the left limbs, and Babinski (+). The National Institutes of Health Stroke Scale score at admission was 13. The patient had a history of diabetes and smoking. He underwent no regular physical examination and did not receive any antiplatelet or anticoagulant drugs. Furthermore, he underwent non-contrast head CT (NCCT), computed tomography angiography (CTA), and CT perfusion (CTP). CTA of the head and neck showed a membrane-like structure in the posterior wall of the right common carotid artery (Fig. 1a) and complete occlusion of the right middle cerebral artery (MCA). The patient then received 0.6 mg/kg intravenous thrombolysis bridging mechanical thrombectomy (MT). Digital subtraction angiography (DSA) revealed a shelf-like filling defect in the carotid bulb, consistent with a CaW (Fig. 1b) and complete right MCA occlusion (Fig. 1c). Immediate MT was followed by retrieval of one red thrombus. DSA revealed that blood vessels were not reoccluded, and the MCA system achieved eTICI3 reperfusion (Fig. 1d).

After the mechanical embolus retrieval, 40 mg of enoxaparin sodium was subcutaneously administered twice daily for anticoagulation for 10 days, followed by oral administration of rivaroxaban (15 mg/day) for the next 20 days, and before 3 days of carotid endarterectomy (CEA), clopidogrel (75 mg/day) was orally administered for antiplatelet combined anticoagulant therapy. Since MT completion, oral treatment with atorvastatin (40 mg/day) was started. When the patient underwent carotid endarterectomy, which exhibited thrombus formation where the web protruded, despite anticoagulant combined antiplatelet therapy. We obtained a thrombus from the rostral CaW aspect after CEA.

**Fig. 2:**
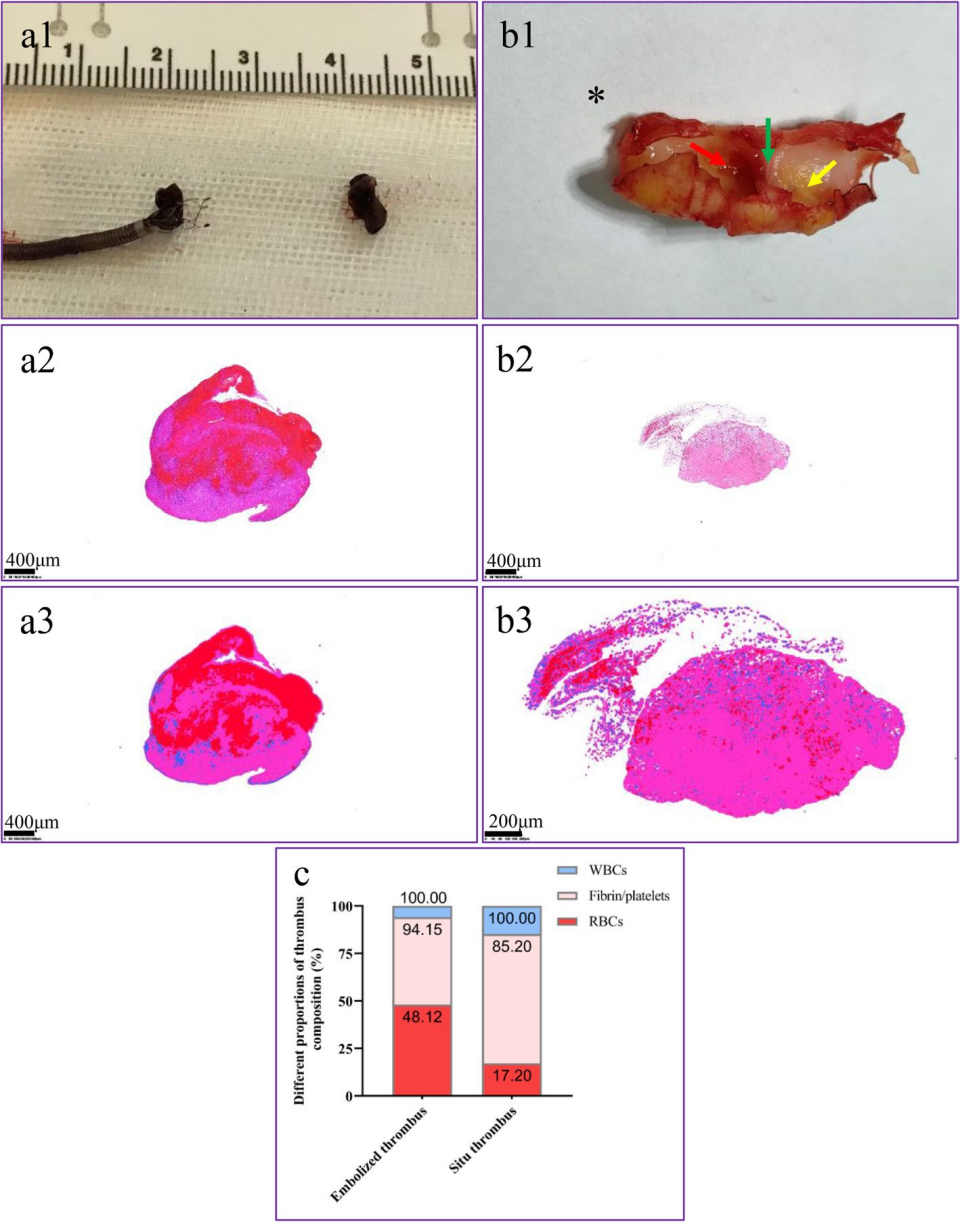
Gross specimen (a1, b1), H&E staining (a2, b2) and clot analysis (a3, b3, c). (a1) Retrieved clots from the right MCA, (b1) CaW superimposed thrombus after CEA. (a2, b2) H&E-stained sections of retrieved cerebrovascular embolized clots and in situ thrombi magnified 40x. H&E staining showing red blood cells (red), fibrin/platelets (pink), and white blood cells (blue). (a3,b3, c) The relative proportion of red blood cells, fibrin, and platelets was quantified using the Orbit Imaging Analysis Software based on H&E staining, in situ thrombi magnified 100x. *, cranialis; red arrow, thrombus along the web; green arrow, web; yellow arrow, fatty streak

The retrieved thrombi (Fig. 2 a1,b1) were immediately fixed in 10% paraformaldehyde and submitted to hematoxylin and eosin (H&E) histological staining (Fig. 2 a2,b2). According to H&E staining, histological quantification of each component was performed using the Orbit Imaging Analysis Software (www.Orbit.bio) following the standard operating procedure (Fig. 2 a3,b3) [7].

The histopathologic qualitative analysis of the clots from various sites in this patient showed a particular appearance. That of the embolized thrombus corresponded to a mixed thrombus with a smooth surface of red blood cells (RBCs) compatible with fibrin composition (Fig. 2 a2), while the in situ thrombus had a “white” clot appearance with an abundant fibrin/platelet apparent surface. RBC components comprised nearly half of the embolized thrombus but were rare in the in situ thrombus. Although the clot included a small number of white blood cells, they accounted for a larger proportion in the in situ thrombus than that in the embolized thrombus. Quantitative thrombus composition analysis of the embolized clot yielded 46.03% fibrin/platelet, 48.12% RBCs, and 5.85% white blood cells; the thrombus in situ yielded 68.0% fibrin, 17.2% RBCs, and 14.8% white blood cells (Fig. 2 c).

## Discussion and conclusions

Herein, we provide a histologic examination of the thrombi obtained from either the CaW in situ during CEA or during embolectomy in the patient with ipsilateral CaW. We hypothesized that there would be compositional differences between CaW in situ and embolized thrombi. The present study showed a different histologic appearance between the thrombus in situ and the embolized thrombus caused by CaW.

The histologic appearance of cerebrovascular embolized clot resembles that of a mixed thrombus primarily containing RBCs and fibrin, whereas that of CaW in situ thrombus appears more like a “white” thrombus composed primarily of fibrin and platelets. Despite its low percentage of white blood cells, the white blood cell component accounted for a larger proportion of the thrombus in situ than other thrombi. However, in situ thrombi were observed to be small at the same time. If thrombus in situ was combined with embolized thrombus as a whole, the entire thrombus also had a mixed clot appearance. Essig et al. found a similar composition within identical thrombemboli by comparing two different slices of a single thrombembolus [8], which supports the slicing method for composition analysis.

An embolized thrombus does not appear to have any unique characteristic histological features on basic H&E staining. The cerebrovascular thrombus was composed of roughly equal parts of RBCs and fibrin/platelets with interspersed white blood cells, consistent with a previous study [9]. The white blood cell composition of the embolized thrombus in our case was minimal, similar to that observed in other clots [10, 11].

Although the architecture of histology of stroke from other etiologies has been studied, the results are inconsistent. Previous studies have suggested that cardioembolic thrombi have higher proportions of fibrin/platelets, fewer RBCs, and more white blood cells than non-cardioembolic thrombi [12–14]. This was consistent with the in situ thrombus composition in the present case. This could be explained by similar mechanisms of hemodynamic changes producing similar thrombotic components. However, other studies on retrieved emboli in large vessel occlusion stroke patients found that patients with strokes secondary to arteriosclerotic emboli were more likely to be platelet-rich than those with cardioembolic strokes [10, 15]. Ahn et al. observed that in arteriogenic clots, platelets covered the fibrin layers or were localized at the clot edge or its periphery, whereas in cardiogenic thrombi, fibrin was the most abundant, and platelets were clustered within fibrin-rich regions [16]. Therefore, it is difficult to infer the relationship between thrombosis and pathological composition.

In the patient with CaW, the mechanism of embolic stroke is unknown. The development of thrombus is believed to be caused by blood flow stagnation along the CaW [4]. Blood flow stasis distal to the CaW results in thrombus formation. This thrombus, when of sufficient size, dislodges and embolizes intracranially. The impact of disturbed flow on thrombogenesis has long been debated [9]. In post-stenotic regions, blood flow stagnation and low wall shear stress are more prominent [17, 18]. Local flow stasis in the web pocket may be responsible for thrombus development and cerebral embolism. Theoretically, this is a situation that promotes the development of red thrombus [2, 19]. In the present case, the histologic features of thrombus in situ are similar to a “white” clot and that of embolized thrombus is similar to a mixed clot. Let us prefer to the pathophysiological process of thrombosis that originates from platelet accumulation caused by endothelial dysfunction of the CaW, similar to the thrombosis mechanism of cardioembolic etiology [20].

Previous studies have focused on the histology of web-induced thrombosis, primarily on secondary thrombosis [9]. Although this is a case report of a single patient, the findings that fibrin/platelet is the major component of CaW in situ thrombi and the embolized thrombus is a mixed thrombus may provide a new explanation for thrombus formation and CaW development.

Our study had some limitations. First, advanced staining techniques, including immunostaining approaches, were not used in this report to investigate different components, such as platelets and vWF. Second, no pathological examination of the CaW was performed.

In conclusion, we present a patient with acute ischemic stroke of the CaW treated with MT and anticoagulants combined with antiplatelet therapy followed by CEA. Histological staining showed significant histological differences between CaW in situ and embolized thrombi. These findings provide a histologic rationale for the new hypothesis that CaW promotes thrombus formation. However, further research is needed to distinguish clot composition and the exact thrombosis mechanism of CaW. CaW is prone to generating new unstable thrombi and could theoretically cause an embolic stroke. Therefore, early surgical intervention should be advocated for prevention.

## Data Availability

All data are available in the manuscript.

## Abbreviations

CaW: Carotid web
CEA: Carotid endarterectomy
CTA: Computed tomography angiography
CTP: CT perfusion
FMD: Fibromuscular dysplasia
H&E: Hematoxylin and eosin
MCA: Middle cerebral artery
MT: Mechanical thrombectomy
NCCT: Non-contrast head CT
RBC: Red blood cell

## References

[1] Kim SJ, Nogueira RG, Haussen DC (2019). Current understanding and gaps in research of carotid webs in ischemic strokes: a review. JAMA Neurol.

[2] Joux J (2014). Carotid-bulb atypical fibromuscular dysplasia in young afro-Caribbean patients with stroke. Stroke.

[3] Joux J (2016). Association between carotid bulb diaphragm and ischemic stroke in young afro-Caribbean patients: a population-based case-control study. Stroke.

[4] Choi PM (2015). Carotid webs and recurrent ischemic strokes in the era of CT angiography. AJNR Am J Neuroradiol.

[5] Sumi T (2010). Disturbed blood flow induces erosive injury to smooth muscle cell-rich neointima and promotes thrombus formation in rabbit femoral arteries. J Thromb Haemost.

[6] Haussen DC (2017). Carotid web (intimal Fibromuscular dysplasia) has high stroke recurrence risk and is amenable to stenting. Stroke.

[7] Stritt M, Stalder AK, Vezzali E (2020). Orbit image analysis: an open-source whole slide image analysis tool. PLoS Comput Biol.

[8] Essig F (2020). Immunohistological analysis of neutrophils and neutrophil extracellular traps in human Thrombemboli causing acute ischemic stroke. Int J Mol Sci.

[9] Koneru S (2021). Clot composition in retrieved thrombi after mechanical thrombectomy in strokes due to carotid web. J Neurointerv Surg.

[10] Fitzgerald S (2019). Platelet-rich emboli in cerebral large vessel occlusion are associated with a large artery atherosclerosis source. Stroke.

[11] Liebeskind DS (2011). CT and MRI early vessel signs reflect clot composition in acute stroke. Stroke.

[12] Sporns PB (2017). Ischemic stroke: what does the histological composition tell us about the origin of the thrombus?. Stroke.

[13] Boeckh-Behrens T (2016). Thrombus histology suggests Cardioembolic cause in cryptogenic stroke. Stroke.

[14] Liao Y (2020). Differences in pathological composition among large artery occlusion cerebral thrombi, Valvular heart disease atrial thrombi and carotid Endarterectomy plaques. Front Neurol.

[15] Brinjikji W (2017). Correlation of imaging and histopathology of thrombi in acute ischemic stroke with etiology and outcome: a systematic review. J Neurointerv Surg.

[16] Ahn SH (2016). Histologic features of acute thrombi retrieved from stroke patients during mechanical reperfusion therapy. Int J Stroke.

[17] Cheng C (2006). Atherosclerotic lesion size and vulnerability are determined by patterns of fluid shear stress. Circulation.

[18] Malek AM, Alper SL, Izumo S (1999). Hemodynamic shear stress and its role in atherosclerosis. Jama.

[19] Jackson SP (2011). Arterial thrombosis--insidious, unpredictable and deadly. Nat Med.

[20] Akoum N (2016). New perspectives on atrial fibrillation and stroke. Heart.

